# Supplementary material for: Complexome profiling of the Chlamydomonas psb28 mutant reveals TEF5 as an early PSII assembly factor
Source: Plant Cell. 2025 Mar 17;37(6):koaf055. doi: 10.1093/plcell/koaf055 (PMC12164590; doi:10.1093/plcell/koaf055)
Supplement: koaf055_Supplementary_Data [file koaf055_supplementary_data.zip › psb28_interactive.zip!/plots/intensity/qualityPlots/peptides/_Cre13.g602450.t1.2.html]

### Cre13.g602450.t1.2

Trivial name:   
SeqCoverage [median]: 30.40  
Mass: 23.56 kDa  
  
 

| Sequence | MissedCleavage | Mass [Da] | wtIntensity | muIntensity | Score |
| --- | --- | --- | --- | --- | --- |
| AAGSALLDSLIR | 0 | 1185.67 | 6125.91 | 4361.56 | 158.79 |
| ASLFTASSK | 0 | 910.48 | 4353.51 | 6741.44 | 129.42 |
| FIAPIKVAQQR | 1 | 1269.76 | 999.20 | 1483.05 | 65.35 |
| IIIKNVTDFTDPEEK | 1 | 1760.92 | 902.05 | 0.00 | 65.81 |
| NVTDFTDPEEK | 0 | 1293.57 | 5611.43 | 4819.88 | 173.64 |
| SYQLLDSELEAAVEGSGAGSGR | 0 | 2195.03 | 443.56 | 0.00 | 138.55 |
|  |  |  | 18435.6 | 17405.9 |  |
